# Efficacy and safety of the application of extensive ablation in patients with atrioventricular re-entrant tachycardia (a retrospective study)

**DOI:** 10.1038/s41598-021-92935-0

**Published:** 2021-06-28

**Authors:** Guangze Xu, Zhikui Chen, Haiyan Lin

**Affiliations:** Department of Cardiology, Lihuili Hospital of Ningbo University, #57 Xingning Rd, Ningbo, Zhejiang People’s Republic of China

**Keywords:** Cardiology, Interventional cardiology

## Abstract

Radiofrequency catheter ablation (RFCA) has become the standard effective therapy for supraventricular tachycardia, but the reported success rates of ablation have differed across a large number of single-center studies. The main reason for tachycardia recurrence is accessory pathway (Ap)-mediated tachycardia, and the use of the RFCA strategy may be related to recurrence. This study compared the efficacy and safety of two different RFCA strategies for Ap-mediated tachycardia. We compared patients (group M) who underwent RFCA at multiple sites to patients (group S) who underwent RFCA at a single site during the index procedure for Ap-mediated tachycardia. The efficacy and safety were assessed in the two groups. Follow-up was conducted, and the main complications and the incidence of recurrence after RFCA procedures were recorded. Eight hundred eighty-two patients with 898 Aps were enrolled in group S, and 830 patients with 843 Aps were enrolled in group M. The cumulative number of recurrences (rates) in group M and group S at the 1st, 3rd, 6th, 12th, and 24th months after ablation were 4 (0.5%) and 17 (1.9%), p < 0.05; 5 (0.6%) and 27 (3.0%), p < 0.05; 6 (0.7%) and 34 (3.8%), p < 0.05; 6 (0.7%) and 43 (4.8%), p < 0.05; and 7 (0.8%) and 45 (5.0%), p < 0.05, respectively. Complications of chest pain, overactive vasovagal reaction, steam pop, and angina pectoris were rare in both groups. One patient in group M suffered from myocardial infarction before extensive ablation. No valve damage, cardiac tamponade, or other serious adverse events occurred in either group. The extensive ablation strategy reduced the recurrence rate and the need for subsequent ablation of the Ap without increasing the risk of complications.

## Introduction

Radiofrequency catheter ablation (RFCA) has been widely performed in more than 600 hospitals in China since 1991. In 2018, the annual number of RFCA procedures performed in China reached 133,900^1^, of which approximately 50% were conducted to treat paroxysmal supraventricular tachycardia (PSVT). Although the success rate of RFCA for PSVT was higher than that of RFCA for other arrhythmias, 2–5% of the patients experienced recurrence^2–6^, and atrioventricular re-entrant tachycardia (AVRT) cases accounted for the greatest proportion. Accessory pathways (Aps) in the patients with failed initial procedures were successfully ablated during a second or third procedure, suggesting that modification of the ablation strategy might improve the success rate. After altering the ablation strategy by establishing a “bonus” lesion at the same site as or a site similar to that of successfully RF ablated AP, the overall success rate at the 2-year follow-up after the initial ablation of AVRT in our center increased from 95 to 99.2%. This approach may be common practice in most electrophysiology labs, but no previous study of its efficacy and safety has been published. Here, we report the method and our experience.

## Methods

### Study population

A total of 1712 consecutive patients with 1741 Aps were enrolled in this study from 2002 to 2018. The patients were divided into the S group (882 patients with 898 Aps) and the M group (830 patients with 843 Aps) based on the ablation strategy used. Ablation was performed at our hospital using the S method, with a single ablation, before 2011, while ablation with the M method, using multiple ablations, has been performed since 2012. Patients with Ebstein’s anomaly and epicardial bypass tracts were excluded. We also excluded patients with para-Hisian Aps. Preoperatively, patients with the following severe diseases were excluded: Acute myocardial infarction, hemodynamic instability, intracardiac thrombus, significant coagulation dysfunction, severe coronary heart disease without revascularization, symptomatic aortic valvular disease, active infective endocarditis, incompatible mental disorders, and extreme heart failure. All the patients signed informed consent forms prior to the EP procedure consenting to participate and be contacted for follow-up after the ablation. Our study was approved by the Ethics Committee of Lihuili Hospital, and all methods were performed in accordance with the relevant guidelines and regulations.

### Electrophysiology study, mapping, and ablation strategy

EP studies were performed after all cardioactive drugs had been discontinued for at least five elimination half-lives. The Seldinger method was used to puncture the femoral vein in all patients. Under X-ray guidance (UNIQ FD20, Koninklijke Philips, the Netherlands), four 6F multielectrode mapping catheters with spaces of 2–4–2 mm between the electrodes were introduced percutaneously through the right femoral or jugular veins. The electrodes were placed in a stable position to record the bundle, lateral right atrium, right ventricular apex, and coronary sinus (CS) activities as required. Anticoagulant treatment was started before the left ventricle was accessed (heparin, 100-U/kg iv bolus, followed by a 1000-U/h infusion) to maintain an activated clotting time (ACT) between 250 and 300 s. The long introducer sheath was rinsed with heparin saline during the EP procedure.

A 4-mm-tip non-irrigated ablation catheter (⌀7 Fr) from one of various manufacturers (Biosense Webster, CA, USA; Abbott, MN, USA; Triguy, Shenzhen, China; or Synaptic, MN, USA) was randomly selected. No procedures at our center were performed with an irrigated catheter. A 0.5- to 500-Hz filter was set for the unipolar recording of the electrocardiogram of the endocardium, while a 30- to 150-Hz filter was used for the bipolar recording. Three surface electrocardiographic leads were displayed and recorded simultaneously with intracardiac electrograms at a paper speed of 100 mm/s. High amplification in the range of 0.1 mV/cm was used to record the potentials. A Mac/Cardio-clab EP recorder (GE Healthcare, Chi, USA), Stockert Ep-shuttle RF ablation apparatus (Biosense Webster, CA, USA), and EP-4 cardiac electrophysiological stimulators (St. Jude Medical, MN, USA) were used in the EP procedure. Long introducer sheaths (Swartz R0, Abbot) were routinely used to facilitate mapping along the tricuspid annulus. For 3D electroanatomic mapping, CARTO3 (V4.3.5.68; Biosense Webster, Irvine, CA, USA) was used for a few of the patients.

All selected patients underwent a detailed EP evaluation at our institution, and the diagnostic criteria for AVRT/Wolff-Parkinson-White syndrome (WPW) reported in the 3rd edition of Clinical Cardiac Electrophysiology Techniques and Interpretation were used^7^. Multiple atrioventricular Aps were defined as the two earliest excitatory sites located more than 2 cm apart or bilaterally.

Most of the procedures were performed primarily using the transaortic (retrograde) approach, but the transseptal approach facilitated mapping of the atrial aspect of the mitral annulus in patients in whom mapping and/or ablation of the ventricular aspect failed. Some patients chose the transseptal approach because it offered rapid postprocedure discharge. The presence and location of an Ap were confirmed based on the standard criteria. The stimulation, recording and mapping methods used in our laboratory and the definitions used have previously been described in detail^8–10^.

A power of 30–50 W targeting a temperature of 50–60 °C was used for the procedure. If Ap conduction was not blocked within 5 s, energy delivery was discontinued, and the mapping criteria and catheter contact were re-examined. If the Aps were blocked within 5 s (loss of anterograde and retrograde conduction), energy delivery was continued for up to 90 s. The impedance, atrial/ventricular (A/V) electrogram, and catheter position were continuously monitored during the ablation procedure. Complete bidirectional blockage of the Aps was set as the end point of the ablation procedure. Electrical cardioversion was applied for patients with atrial fibrillation to evaluate the retrograde conduction of Aps.

**Figure 1:**
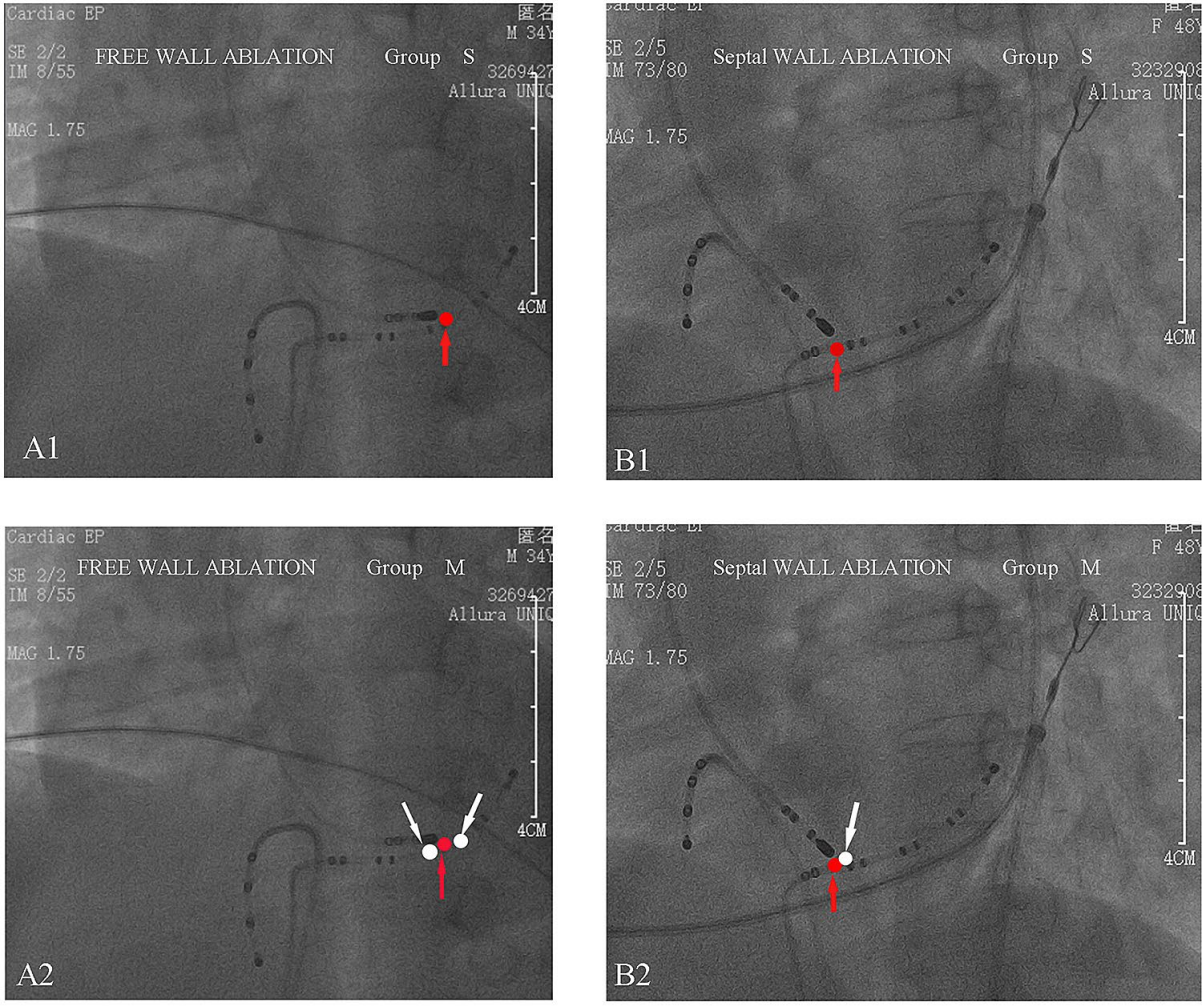
(**A1**) Shows the localization of the ablation site for a free wall Ap in group S (the red arrow refers to the initial site of successful ablation). (**A2**) Shows additional ablation sites on both sides adjacent to the initial site of successful ablation for the free wall Ap (the white point refers to the additional ablation site). Septal wall Aps in group M were treated with an additional ablation on the side adjacent to the first successful site and away from the septum (**B1**,**B2**).

**Figure 2:**
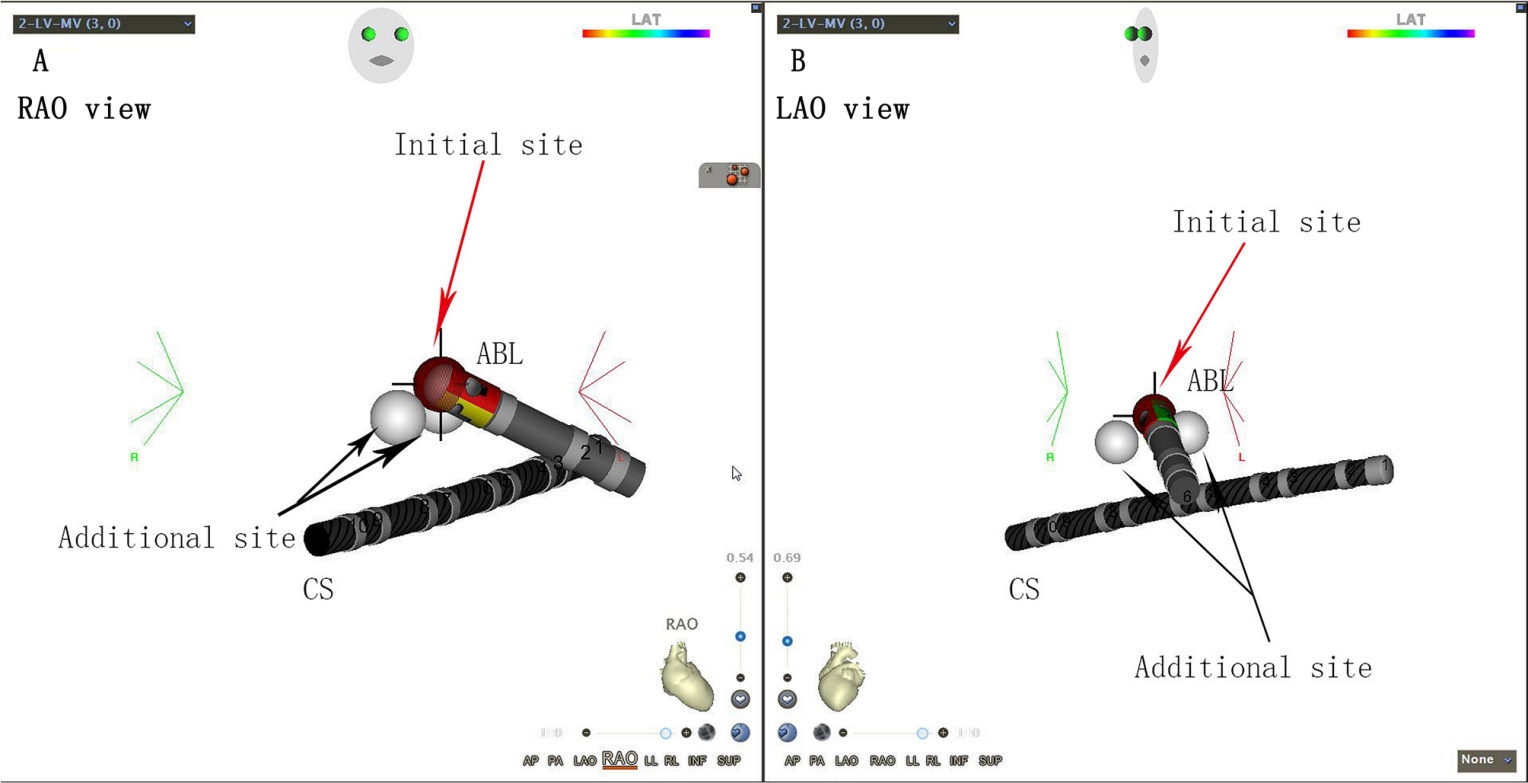
3D electroanatomic mapping (CARTO3 V4.3.5.68; Biosense Webster, Irvine, CA, USA) for Ap ablation. The red point refers to the initial ablation site, and the white point shows the additional site. *CS* coronary sinus, *ABL* ablation catheter.

The initial bidirectional blockade of Ap conduction was set as the end point of the procedure in group S. Free wall Aps in group M were addressed with additional ablation for 60 s on either side of the site of initial success (within the interelectrode space). Aps of the septal wall in group M were treated with additional ablation for 60 s on the side adjacent to the first successful site but away from the septum. Figures 1 and 2 show the strategies used in the two procedures.

All patients were routinely monitored for 24 h after the EP procedure. The puncture site in the femoral vein was compressed for two hours, while the femoral artery was compressed for 6 h. All procedures were performed by an experienced electrophysiologist, and all patients signed informed consent forms.

### Follow-up strategy

Symptoms were the main indication of recurrence after discharge, and routine ECG checks of all patients were conducted during the follow-up period to help identify asymptomatic cases of recurrence. The main complications and incidence of recurrence after EP procedures were recorded. Follow-up was conducted at the 1st, 3rd, 6th, 12th, and 24th months after ablation via clinical intervention or a telephone call after discharge. The longest observation period was set at 24 months or the time of relapse. Interphase of the last two episodes of AVRT before the EP procedure was recorded as the frequency of tachycardia. The observed interphases may have been related to our observational endpoint, and this possibility dictated our follow-up period. In some cases, the interphase of AVRT may exceed 2 years; obviously, in such situations, recurrence might not be observed. However, this was not an issue in patients with preexcitation. When preexcitation of the body surface ECG reappeared, recurrence was identified. Therefore, in this manuscript, we set the interphase of the last two episodes of WPW as 0 months. We attempted to reduce the medical expenses associated with the additional procedure to avoid the loss of information from patients with recurrence to the greatest extent possible. The cumulative recurrence rates at the 1st, 3rd, 6th, 12th, and 24th months after ablation and the incidence of perioperative complications, including chest pain, overactive vagal reflex, thromboembolic events, pericardial tamponade, tissue vaporization (steam pop), valve damage, angina pectoris, and myocardial infarction, were observed.

### Statistical analysis

Normally distributed continuous data are presented as means ± standard deviations and were compared using independent-sample t tests, and nonnormally distributed continuous data are presented as the medians (Q1, Q3) and were compared using Mann–Whitney U tests. Numerical data are reported as counts and rates, and Fisher’s test or Pearson’s *X*^2^ test was used for comparisons. Recurrence rates were estimated with the Kaplan–Meier method. Cox proportional hazards models were used to determine factors associated with the risk of recurrence. SPSS 23 software was used for the statistical analysis, and p = 0.05 was set as the level of significance for two-sided tests.

### Ethical approval

All patients signed informed consent forms prior to the EP procedure. The study was approved by the Ethics Committee of Lihuili Hospital of Ningbo University. All methods used in our study were conducted in accordance with relevant guidelines and regulations.

## Results

### Baseline characteristics

Preoperative and intraoperative baseline data were compared (Table 1) as described in other reports^11–15^ and included age, sex, Ap location, number of Aps, frequency of tachycardia episodes, number of initial ineffective ablations, catheter approach, concomitant coronary artery disease (CAD), concomitant atrial fibrillation, concomitant heart failure (ejection fraction (EF)% < 35%), and other factors related to the prognosis and complications. Eight hundred eighty-two patients with 898 Aps were included in the S group; they ranged in age from 10 to 80 years (mean age, 47 ± 16 years) and consisted of 365 females and 517 males. Among all 898 Aps, 688 (76.6%) were left-sided, 210 (23.4%) were right-sided, and 164 (18.3%) were septal. The mean age of the patients in group M was 48 ± 17 years, with females accounting for 40.7% of patients. Eight hundred forty-three Aps were observed in group M, with 642 (76.2%) Aps on the left, 149 (16.7%) involving the septum, and 201 (23.8%) on the right. The median frequency of tachycardia was 1 (1, 3) month in group S and 1 (1, 2) month in group M. Ablation of the atrial aspect was successful in 28.6% of the patients in group S and 27.4% of the patients in group M. Additionally, 6.2% of the patients in group S and 7.2% in group M underwent an EP procedure with a 3D mapping system. In group S, 1.4% of the patients had a history of coronary heart disease, 2.4% had preexcitation with atrial fibrillation, and 0.2% had an EF < 35%. In group M, 0.8% of the patients had a history of coronary heart disease, 1.5% had preexcitation with atrial fibrillation, and 0.4% had an EF < 35%. No significant differences in any presented factor were observed between the two groups.

**Table 1:** Main clinical features of patients stratified by different ablation methods.

Basic characteristics	Group S (882 patients)	Group M (830 patients)	p
Age (years)	47 ± 16	48 ± 17	0.119
Sex (female)	365 (41.4%)	338 (40.7%)	0.781
Ap number	898	843	
Multiple Aps	16 (1.8%)	13 (1.6%)	0.691
Initial ineffective ablation	1.72 ± 0.80	1.68 ± 0.83	0.379
Left Aps	688 (76.6%)	642 (76.2%)	0.822
Septal wall	123 (13.7%)	119 (14.1%)	0.801
Free wall	565 (62.9%)	523 (62%)	0.706
WPW	67 (7.5%)	61 (7.2%)	0.857
Right Aps	210 (23.4%)	201 (23.8%)	0.822
Free wall	169 (18.8%)	171 (20.3%)	0.441
Septal wall	41(4.6%)	30 (3.6%)	0.288
WPW	23 (2.6%)	19 (2.3%)	0.676
Episode frequency (months)	1 (1, 3)	1 (1, 2)	0.885
**Ablation site**			
Atrial aspect	257 (28.6%)	231 (27.4%)	0.572
Ventricular aspect	641	612	–
Transseptal approach	13 (1.5%)	15 (1.8%)	0.227
3D mapping	55 (6.2%)	60 (7.2%)	0.41
WPW with AF	22 (2.4%)	13 (1.5%)	0.177
CAD* history	13 (1.4%)	7 (0.8%)	0.227
EF* < 0.35%	2 (0.2%)	3 (0.4%)	0.607

*EF ejection fraction; *CAD* coronary artery disease.

### Postprocedure characteristics

#### Efficacy analysis

**Figure 3:**
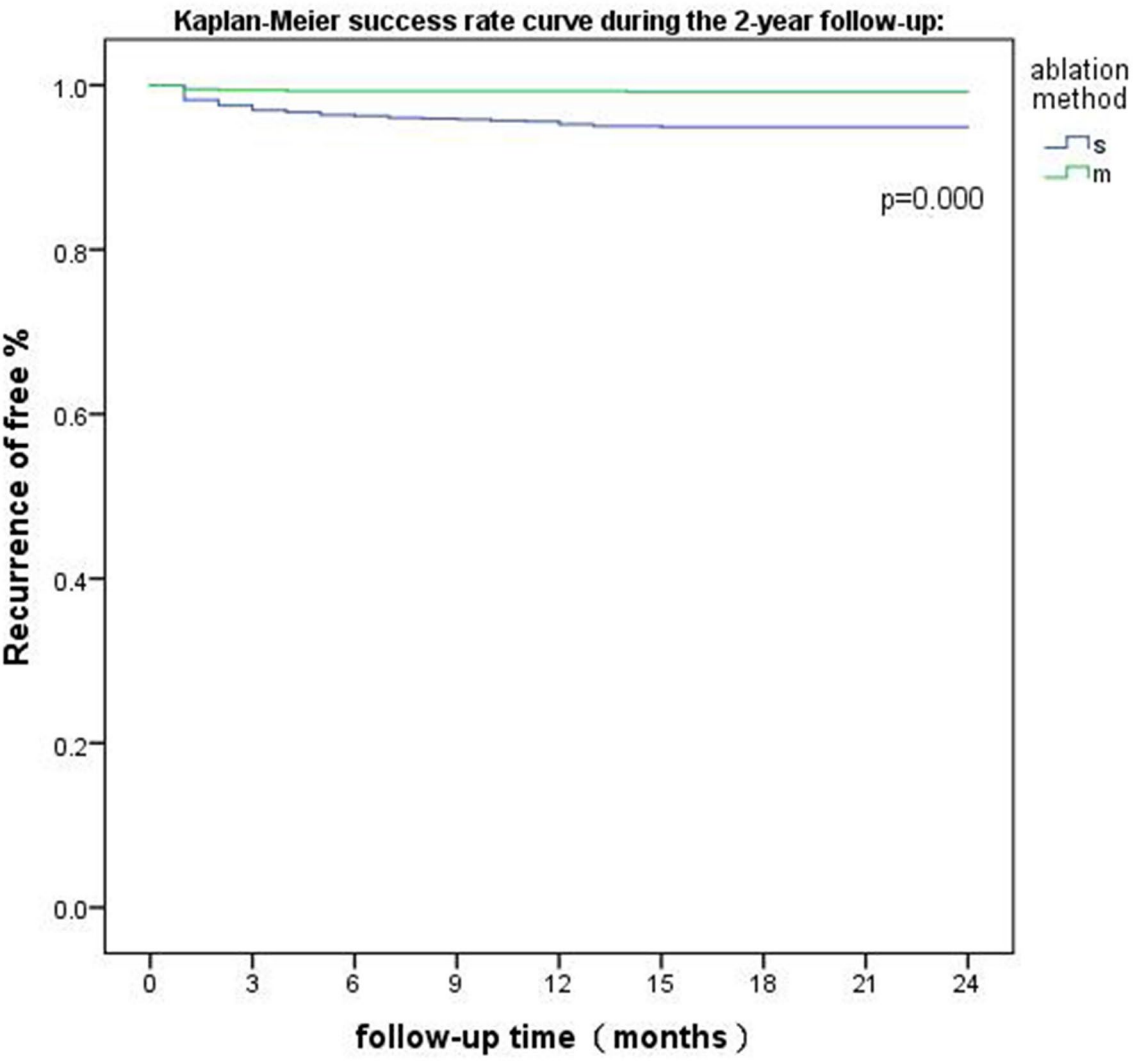
The survival curves of the two groups during the 2-year observation period. The cumulative recurrence rate at every observation time point was significantly lower in group M than in group S. Group M showed an advantage in terms of a high ablation success rate (log-rank test (Mantel-Cox) p value < 0.05).

The immediate success rate in both groups was 100%. All patients were observed for 1–24 months, with a median follow-up of 24 months. Forty-five cases of recurrence were identified in group S, and 7 cases were identified in group M. The cumulative recurrence rate in each observation period was significantly lower in group M than in group S, and the cumulative number of recurrences (rates) in group M and group S at months 1, 3, 6, 12 and 24 was 4 (0.5%) and 17 (1.9%), p < 0.05; 5 (0.6%) and 27 (3.0%), p < 0.05; 6 (0.7%) and 34 (3.8%), p < 0.05; 6 (0.7%) and 43 (4.8%), p < 0.05; and 7 (0.8%) and 45 (5.0%), p < 0.05, respectively. More than 90% of the recurrences occurred within 1 year after ablation in both groups. The cumulative recurrence rate in group M was lower than that in group S during the entire follow-up period (Table 2 and Fig. 3). Among all 52 Aps that recurred, 19 were left-sided Aps, and 33 were right-sided Aps (Table 3).

**Table 2:** Cumulative recurrence events after ablation.

Recurrence time	No. of patients (%)		p
	Group S	Group M	
a. Immediately	0	0	–
b. 1 month	17 (1.9%)	4 (0.5%)	p < 0.05
c. 3 months	27(3.0%)	5 (0.6%)	p < 0.05
d. 6 months	34 (3.8%)	6 (0.7%)	p < 0.05
e. 12 months	43 (4.8%)	6 (0.7%)	p < 0.05
f. 24 months	45 (5.0%)	7 (0.8%)	p < 0.05

Separate Cox proportional hazards models were used to assess the risk of recurrence, and 2 predictors, namely, the ablation method and Ap location, were independent significant predictors (p < 0.05). Ablation of right-sided Aps was associated with a 6.16-fold increased risk of recurrence compared to ablation of left-sided Aps. Complementary ablation reduced the risk of recurrence by 83.8% (Table 4).

**Table 3:** Characteristics of recurrence.

Ap location of recurrence	No. of patients (%)		p
	Group S	Group M	
Right side	16	3	p < 0.05
Left side	29	4	p < 0.05

**Table 4:** Stepwise multivariate Cox proportional hazards model: recurrence.

Predictor	OR	95% CI	p
Ap on the right side (19/52)	1.835	(1.043, 3.228)	0.035
Ablation in group M	0.162	(0.073, 0.36)	< 0.05

#### Analysis of procedure safety (Table 5*)*

**Table 5:** Main complications.

Complication type	No. of patients (%)		p
	Group S	Group M	
a. Chest pain	47 (5.3%)	51 (6.1%)	0.468
b. Angina	3 (0.3%)	1 (0.1%)	0.347
c. Overactive vagal response	19 (2.2%)	15 (1.85%)	0.607
d. Pericardial effusion	11 (1.2%)	9 (1.1%)	0.754
e. Myocardial infarction	0	1 (0.1%)	0.302
f. Steam pop	3 (0.3%)	2 (0.2%)	0.707
g. Pulmonary embolism	1 (0.1%)	0	0.332
h. Deep vein thrombosis	2 (0.2%)	1 (0.1%)	0.559

Forty-one patients in group S (51 in group M) developed ablation-related chest pain, which was relieved immediately by stopping the delivery of energy. Three patients (1 in group M) complained of chest tightness with transient changes in ST-T morphology on electrocardiography (ECG). One patient in group M complained of chest pain in the precardiac area during the initial energy delivery; complication with continuous typical ST-segment elevation was confirmed, and instant coronary angiography confirmed a coronary artery occlusion located at the site of the ablation target. No myocardial infarction occurred in group S. No significant difference in the incidence of excessive vagal response was observed between the two groups (19 (2.2%) vs 15 (1.85%), p = 0.607). Steam pops occurred infrequently in both groups (3 (0.3%) vs 2 (0.2%) p = 0.707). Only a few patients in the two groups presented a small amount of pericardial effusion on a routine ultrasound conducted after the EP procedure (11 (1.2%) vs 9 (1.1%), p = 0.754). One patient in group S developed a pulmonary embolism (confirmed by CT angiography) after vascular compression and strict movement restrictions of the lower limb, with sudden dyspnea accompanied by a decrease in blood pressure; no pulmonary embolisms occurred in group M. Only a few patients in both groups suffered from lower extremity venous thrombotic-associated edema (2 (0.2%) vs 1 (0.1%), p = 0.559). No cardiac tamponade, peripheral arterial embolization, valvular injury, or perioperative deaths occurred in either group.

## Discussion

RFCA is an effective, curative and widely used therapy for AVRT. Initially, successful RF ablation is generally persistent, but late recurrence of Ap conduction after single-point ablation is not uncommon. Anatomical variation of Aps and the ablation strategy are suspected to be associated with postablation recurrence.

Most insertions of Aps were reported with sizes of 1 to 3 mm and close to the mitral annulus. Theoretically, complete blockade of most Aps could be achieved by single-point. However, the insertion is not always accurately located due to unique anatomical factors, such as a wide Ap or a dendritic^16^ insertion far from the valve annulus, resulting in incomplete bypass damage and recurrence. Mccleland et al.^17^ reported a group of bypass ducts in which 18% required ablation within the range of 3 cm along the CS to achieve a completely bidirectional block. In addition, 60–80% of Aps are oblique across the valve annulus^18,19^, which would probably result in problems with the localization of the insertion site of an Ap from an improper aspect of the annulus. In this situation, the site of earliest ventricular activation recorded from the atrial aspect of the annulus does not always correspond to the atrial insertion site and vice versa. The transaortic approach is a routine way of mapping anterograde Ap activation, while the transseptal approach is applicable for mapping retrograde Ap activation, and they are always complementary in mapping and ablation. In some cases, alteration of the catheter approach to the other aspect of the annulus may help clarify the obscure potential.

It is not always possible to distinguish atrial electrogram from ventricular electrogram, and pseudodisappearance of atrial electrogram in the process of mapping leads to unrecognized EVA and EAA phenomena and increases the difficulty of locating the Ap insertion site, which is also considered a factor involved in relapse after single-target ablation. In addition, EAA and EVA are sometimes widely distributed, which increases the difficulty of mapping and ablation and suggests the possibility of a wide pathway.

The initial success of immediate ablation indicates that the catheter tip is positioned at or near the insertion site of the Ap, but whether the bypass has been irreversibly damaged cannot be determined. According to reports^20,21^, the effective ablation depth of conventional 4-mm-tip ablation catheters is 4–6 mm, and we selected a distance within the interelectrode space of the CS catheter (< 4 mm) next to the initial successful target along the CS as the site of the additional ablation. Theoretically, continuous fusion and effective heat penetration in target tissues can be achieved. Therefore, we proposed performing an additional ablation near the first successful target to reduce the recurrence rate. Using this ablation strategy, the success rate of AVRT ablation at our center has been significantly improved.

Our study showed a higher recurrence rate after ablation of right free wall and septal Aps than after ablation of left free wall Aps, similar to previous reports^11,15^. These findings can be explained by the increasingly well-recognized target-dependent differences in the ease of mapping and the effectiveness of tissue heating. Catheter shift reduces the effective damage depth of the target site and hinders further effective heat diffusion. We assumed that performing an additional ablation in the vicinity of the initial successful site was advantageous for distributing the energy to the correct depth from the side.

We routinely reference the coronary sinus catheter (with projection angles RAO 30° and LAO 45°) to estimate the distance and direction of the movement of tip of the ablation catheter. By pulling or pushing to rotate the handle of the ablation catheter as needed, the catheter tip can undergo a minor shift to the site for extensive ablation. Theoretically, a 3D mapping system would be a precise way to localize sites for both initial and extensive ablation. In our study, patients who were treated under the guidance of 3D mapping were scattered sporadically in both groups, and there was no significant difference in distribution. Thus, this issue did not influence the results of the study. Because the ablation catheter was in similar way in all patients, we always obtained a similar distance shift from the initial ablation site under both the 3D mapping and fluoroscopy conditions in many extensive ablation procedures.

New mapping systems that work with artificial intelligence (AI) and an improved energy delivery catheter (e.g., irrigation catheter) are expected to improve the accuracy of Ap localization and increase the stability of the catheter. This technique will enlarge the ablation lesions and reduce the rate of recurrence but entail a higher cost borne by the national insurance policy of China. Conventional mapping and catheter manipulation under fluoroscopic guidance is still the main method for the EP assessment of PSVT in most developing countries, and a modification of the ablation strategy to reduce the recurrence rate is necessary.

Additional ablation entails added X-ray exposure and EP procedure times. Theoretically, an increased risk of complications, including myocardial infarction, myocardial perforation, and cardiac tamponade, may exist, particularly for patients undergoing atrial aspect ablation. Repeated subvalvular ablation increases the risk of steam pop, and repeated catheter manipulation may increase the risk of thrombosis, embolism, and valve damage. All of these factors must be considered. As our study showed, the incidence of complications was similar in both groups, and no complications occurred during the additional ablation in group M. The incidence rates of various complications at our center were lower than those reported by other EP centers^3,11,22^. We suggest that if the initial mapping and ablation are successful, the level of increased risk associated with performing an additional ablation is acceptable. But we don't propose multiple ablations from the get go of EP procedure, for most patients got the single point ablation success. Diagnostic part is more important part of the procedure as it determines the need of minimal ablation in part mainly to reduce complication and high success rate.

### Limitations

The use of fluoroscopy alone would allow the random delivery of RFCA to the same region, but there is no real reference, either anatomical or electrical, that would be reproducible on an accurate site. The use of a 3D mapping system would provide spatial resolution and would help to make the study more convincing. We hope to conduct a further study that includes more patients with good enough economic conditions to afford 3D system-guided AP ablation in the future to remove doubt about the accuracy of localization. In our retrospective study, data were collected from different periods, leading to inevitable bias in the two groups and possibly affecting the results. Longer ablation times, increased operator experience and technological advances may be the reasons for the improved outcomes in group M. The setting of a single-point ablation time was based on our previous ablation practice and reports from other centers and was considered to be safe and to meet ethical requirements. If sufficient evidence regarding the safety of prolonged-duration ablation at a single site emerges in the future, we will consider establishing a control group in which extensive ablations (90″, 60″, 60″) at the same original site will be performed. A prospective randomized multicenter study is needed to verify the results.

## Conclusions

Compared to conventional ablation, the additional ablation decreased recurrence rate significantly without increasing the risk of complications. Although understanding of accessory pathway course, identification of accessory pathway potential and slant are crucial elements for determining the correct site of ablation, the results of this study may serve as a reference for clinicians considering therapeutic options for some patients, when the site of AP insertion is not clear enough. It, however, suffered from an intrinsic limitation as a retrospective study. Thus, there is need to compare with a prospective randomized multicenter study to test the hypothesis improve the scientific validity.
